# Toward Lithium Recovery Using Modular (and Membraneless) Phase Separation and Extraction (MPSE) Technology with Ionic Liquid (IL) Solvents: Effect of Coatings

**DOI:** 10.1021/acs.langmuir.5c06615

**Published:** 2026-03-28

**Authors:** Aigerim Baimoldina, Fan Yang, Yihan Song, Jad George Touma, Francis Chukwunta, Matthew Coblyn, Cliff Kowall, Goran Jovanovic, Lei Li

**Affiliations:** † Department of Chemical & Petroleum Engineering, 6614 University of Pittsburgh, Pittsburgh, Pennsylvania 15261, United States; ‡ School of Chemical, Biological and Environmental Engineering, 2694 Oregon State University, Corvallis, Oregon 97331, United States; § Consulting Engineer, 4502 Liberty Road, South Euclid, Ohio 44092, United States

## Abstract

In this work, a single-channel slope-plate modular and membraneless phase separation and extraction (MPSE) device was fabricated using 3D printing. The slope-plate was designed with an insertable glass slide to enable surface modification with coatings. Self-assembled monolayers (SAMs) and perfluoropolyether (Zdol) coatings were applied to tailor the surface wettability and enhance the phase separation. Using a model biphasic system of water and hexadecane, both coatings significantly improved the separation efficiency compared with the uncoated device by promoting selective wetting. Building on these results, lithium extraction from a simulated saline solution was investigated using 1-ethyl-3-methylimidazolium bis­(trifluoromethylsulfonyl)­imide ([EMIM]­[NTf_2_]) as the ionic liquid (IL) extractant. The IL effectively extracted Li^+^ from the aqueous phase in bulk extraction, demonstrating its potential for lithium recovery. However, the low interfacial tension between the IL and aqueous phases posed challenges for phase separation. The application of SAM and Zdol coatings effectively mitigated this issue. Overall, integrating tailored surface chemistry with the slope-plate MPSE design shows great promise as an efficient and scalable platform for studying liquid–liquid separation and optimizing ionic-liquid-based extraction processes for lithium recovery from saline sources.

## Graphical abstract

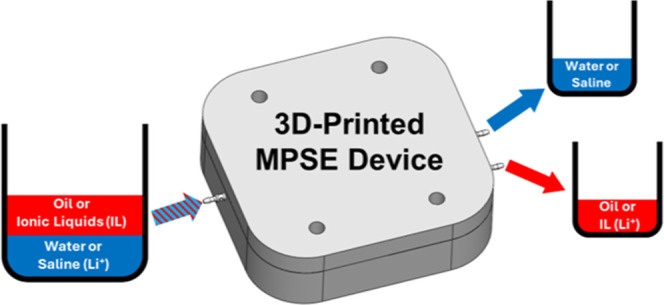

## Introduction

Liquid–liquid extraction (LLE) is a well-established process for liquid–liquid separation in the chemical industry.^1,2^ LLE is the most widely used commercial hydrometallurgical method for the extraction and separation of critical metal ions from aqueous streams. The commercial relevancy of LLE derives from its ability to process large feed volumes while yielding high material purity up to 99.99%.^3,4^ LLE has been used for the extraction of lithium (Li) from brine,^5^ seawater,^6,7^ shale gas produced water,^8^ leaching liquors,^9^ and the separation of Li isotopes.^10^ Additionally, LLE has proved its effectiveness in separating heavy and light rare earth elements (REE),^11^ such as neodymium (Nd) and praseodymium (Pr), from various aqueous feeds.^12,13^ Currently, industrial scale LLE processes are carried out in conventional extraction equipment such as mixer-settlers and centrifugal extractors.^14–16^ These macrounits are characterized by large footprint, long residence times, extensive energy and solvent use, complex unit operations, and high capital and operation costs. Although conventional extraction solvent systems (e.g., tributyl phosphate (TBP)—diethyl succinate—FeCl_3_ for Li extraction) can achieve a good separation, using large amounts of volatile organic solvents cause solvent loss, serious environmental and safety concern, and workers’ health problems.^17^ As a result, it is necessary to find “greener” and safer solvents to replace traditional organic solvents.

Development of microfluidic devices for process intensification (PI) stems from the limitations of conventional macroscale LLE such as low mass transfer efficiency, high solvent and energy consumption, and large hardware footprint.^1,2,18–20^ An increase in solute extraction can be achieved in microfluidic devices^7,10,16,21–24^ and influence of surface tension forces over the gravitational force allows an intensified phase separation in these microscale devices. The main advantages of microfluidic LLE systems present an important increase in process efficiency, controllability, and minimizing of chemical and energy consumption, in addition to ability to number-up microfluidic devices and adapt modular processing.^15,16,25–28^

Recently, a modular, high-throughput, and integrative microfluidic platform called the modular (and membrane-less) phase separation and extraction (MPSE) has been developed.^29,30^ The liquid–liquid phase separation is made possible by a series of microstructures that generate a capillary pressure gradient on the nonwetting phase.^29^ The MPSE technology allows for infinitely scalable process capacity thanks to the numbering-up approach. The fundamentals of the MPSE technical approach are based on the introduction of unconventional surface forces into engineered microscale-based structures that facilitate phase separation and LLE in a multiphase flow. Notably, the movement of a discrete phase (droplets, bubbles) in multiphase flow operations is facilitated with forces arising from the size and shape of the microscale-based architectural features in the device, fluid surface tension, contact angles, chemical composition, and temperature. The interplay of interphase pressure, inertial forces, and drag forces jointly direct the movement of all the fluid phases.

1 $$P_{c}=\sigma \left(\frac{1}{{\mathrm{r̅}}_{x}}+\frac{1}{{\mathrm{r̅}}_{y}}\right)\cdot \cos\theta ,\Rightarrow ,{\frac{\partial \left(\Delta P_{c}\right)}{\partial x}|}_{1/r_{y}=0}=\sigma \cos\theta \cdot \frac{\partial \left(1/{\mathrm{r̅}}_{x}\right)}{\partial x}-\frac{\sigma \sin\theta }{{\mathrm{r̅}}_{x}}\cdot \frac{\partial \theta }{\partial x}+\frac{2\cos\theta }{{\mathrm{r̅}}_{x}}\cdot \frac{\partial \sigma }{\partial x}$$

The Young–Laplace eq (eq 1) accurately captures interrelationships between interfacial tension σ, curvature $\left(1/{\mathrm{r̅}}_{x}\right)$ , and wetting angle of fluids at solid surfaces, θ. While the nature of the surface phenomena originates from intermolecular forces, the surface tension and contact angles represent these forces on a scale much larger than the molecular scale. By extension, the capillary pressure gradient, ∂(Δ*P*_c_)/∂*x*, which is an integral part of the momentum equation in two-phase fluid flow, is also shown in 1D form in eq 1 ( $1/{\mathrm{r̅}}_{y}=0$ for parallel plates). It is obvious that the gradient of the interface pressure is “driven” by gradients of the physical parameters $\partial \left(1/\Delta {\mathrm{r̅}}_{x}\right)/\partial x$ , ∂σ/∂*x*, and ∂θ/∂*x*. In essence, all constitutive parameters of the Young Laplace equation, i.e., *r*, θ, and σ, may play a role in creating interface force. It follows that minimizing (θ) via rendering the surface more energetically favorable to the wetting aqueous phase can increase (Δ*P*_c_) acting on the nonwetting organic phase and therefore improve phase separation.

In addition to creating a series of microstructures to generate the desired capillary pressure gradient,^29^ an alternative method is by controlling the spacing between two flat plates that form the floor and ceiling of the flow channel, what we call the sloped plate channel (Figure 1).^31^ The slope-plate device was intentionally engineered to create a capillary pressure gradient that enabled passive and efficient phase separation without the need for external forces. According to the Young–Laplace relationship, capillary pressure is inversely proportional to the characteristic channel dimension; therefore, the narrow region of the slope plate (right side, Figure 1) generates a higher capillary pressure, while the wider region (left side, Figure 1) produces a lower capillary pressure. This spatial pressure difference establishes a preferential flow pathway that directs the nonwetting phase toward the region of lower capillary resistance. In this design, the aqueous phase acts as the wetting phase relative to the coated surface, allowing it to preferentially occupy the narrow channel, despite the higher capillary pressure. Conversely, the organic phase behaves as the nonwetting phase and is energetically driven toward the wider section, where the lower capillary pressure reduces flow resistance. The geometric asymmetry of the slope plate therefore provides a deterministic mechanism for phase routing while minimizing interfacial instability and undesired phase entrainment. Importantly, this design promotes continuous and stable separation by leveraging fundamental interfacial phenomena rather than relying on density differences. As a result, the narrow portion of the slope plate serves as the outlet for the wetting (aqueous) phase, whereas the wider portion serves as the outlet for the nonwetting (organic) phase, enabling controlled transport and improved separation efficiency at the device level.

**Figure 1:**
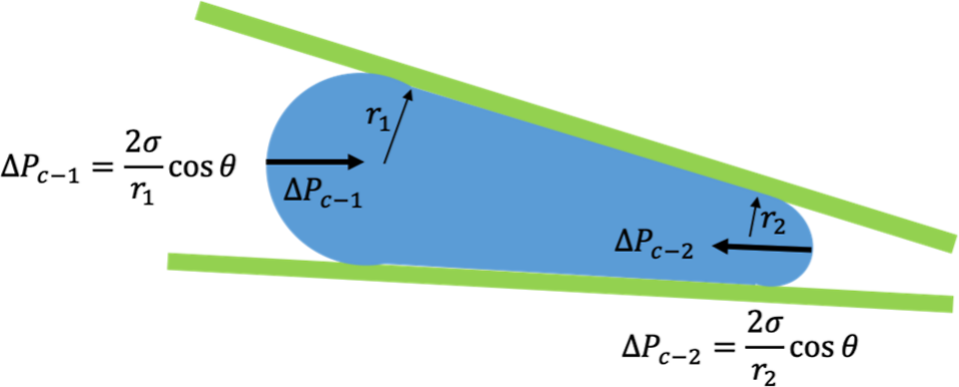
Slope-plate design.

Surface modifications provide further control over the contact angle gradient and can tune the channel to be more wetting or nonwetting for either of the two liquid phases. Additionally, to maximize the separation further, one can apply these surface modifications or coatings to specific regions of the channel. For example, if an oleophilic/hydrophobic coating is applied on the org-exit-port, then the org. phase will be encouraged to go through the org-exit-port while the aqueous phase will be repelled. If a hydrophilic/oleophobic coating is applied on the aqu-exit-port, the aqueous phase will be encouraged to go through the aqu-exit-port while the org. phase will be repelled. In both cases, the separation efficiency is expected to increase. The self-assembled monolayer (SAMs), i.e., chloro­(dodecyl)­dimethylsilane, with a long alkyl chain is ideal for oleophilic/hydrophobic coating.^32^ Meanwhile, a perfluoropolyether (PFPE), commercially known as Zdol, has been demonstrated to be hydrophilic/oleophobic.^33^

In the current work, the single-channel slope-plate MPSE device has been fabricated via 3D printing. To enable coating application, the slope plate is designed to include an insertable glass slide. SAMs and Zdol coatings were applied on the glass slide to enhance the separation efficiency of the slope-plate. The separation of model liquid mixture, i.e., water-hexadecane, using the single-channel MPSE device indicates that both coatings improve the separation efficiency significantly. We then studied the Li extraction from model saline using an ionic liquid (i.e., 1-ethyl-3-methylimidazolium bis­(trifluoromethylsulfonyl)­imide) solvent with the slope-plate. Our research suggests that ionic liquid (IL) is effective in Li extraction from model brine. Although the low interfacial tension between the IL and the aqueous phases makes the separation challenging, the coating approach has been demonstrated to be effective in mitigating the issue.

## Results and Discussion

### Wettability of the Coatings

Chloro­(dodecyl)­dimethylsilane SAMs were used for an oleophilic/hydrophobic coating on glass. The WCA and HCA of glass with and without SAMs coating are shown in Figure 2. Water and hexadecane contact angles of bare glass were both around 10–15° and changed dramatically after SAMs coating. The WCA of the SAMs coated area is around 100° and HCA is around 2°, which indicate simultaneously hydrophobic/oleophilic coating fabrication success.

**Figure 2:**
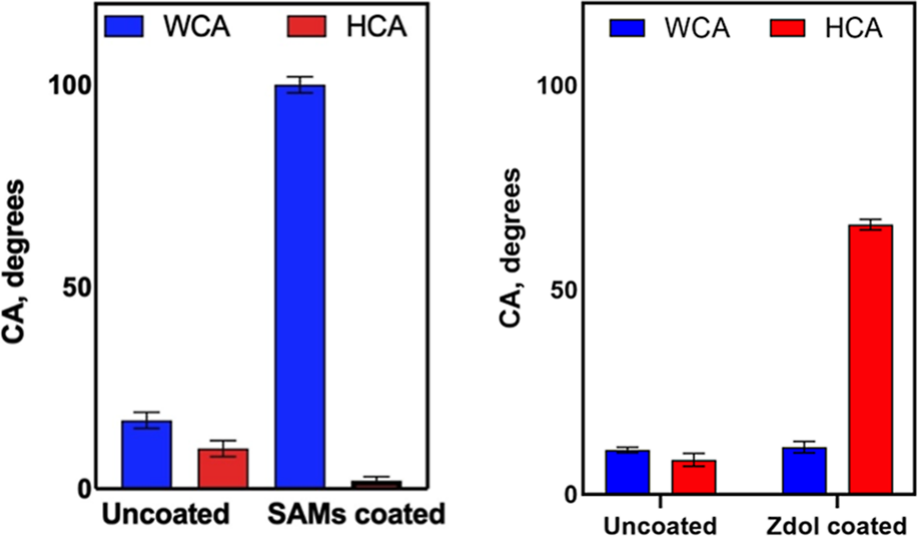
WCA and HCA of bare and coated glass contact angles of bare glass and SAMs (7 nm in thickness) coated glass (left) and Zdol (2.2 nm in thickness) coated glass (right).

To achieve hydrophilicity and oleophobicity, Zdol was coated on the designated location of the glass slides. The WCA and HCA of glass with and without the Zdol coating are shown in Figure 2. Before coating, the WCA and HCA are both less than 10° due to the hydrophilic nature of the glass material, and after coating Zdol on glass, the HCA is increased to above 60°, while the WCA remains unchanged. Having much higher HCA than WCA indicates simultaneously hydrophilic/oleophobic glass is successfully fabricated, and the mechanism of such wetting behavior has been discussed in previous studies.^34,35^

#### Slope-Plate Separation of Water and Hexadecane: Effect of Coatings

Figure 3 (left) shows the opened-up device without the top cover, where two glass slides are sandwiched together by the two 3D-printed spacers to create a sloped channel for fluid flow. The glass slides here enable the coating application. The assembled spacers with glass slides fit into a designated slot of the bottom piece. Two O-rings are used to securely assemble the top cover with four bolts and nuts, as shown in Figure 3 (right). The liquid mixture flows into the device via the inlet tube while separation takes place inside the device. Two outlet streams containing aqueous and organic solutions exit the designated ports, respectively.

**Figure 3:**
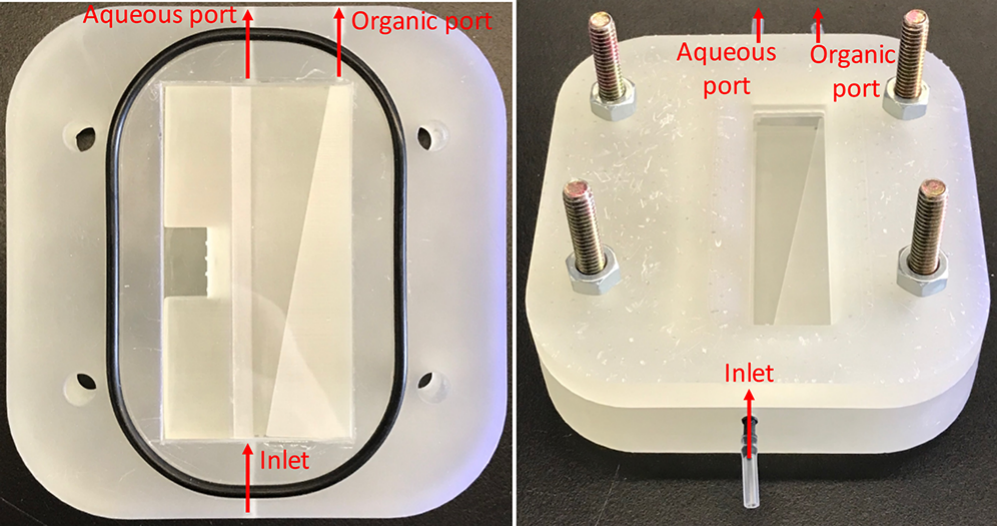
3D-printed slope-plate device. (Left) Opened bottom parts without top cover piece. (Right) Assembled device with a window for monitoring fluid flow. Red arrows indicate flow directions. The channel depth is the shallowest at the aqueous port. (The details on the device geometry and operation condition have been provided elsewhere in our previous work.^31^).

To demonstrate the effectiveness of the single-channel device, a ratio of 50:50 deionized (DI) water and hexadecane liquid mixture feed was introduced to the device using a dual-syringe pump (NE-4000, New Era) (See Figure 4).

**Figure 4:**
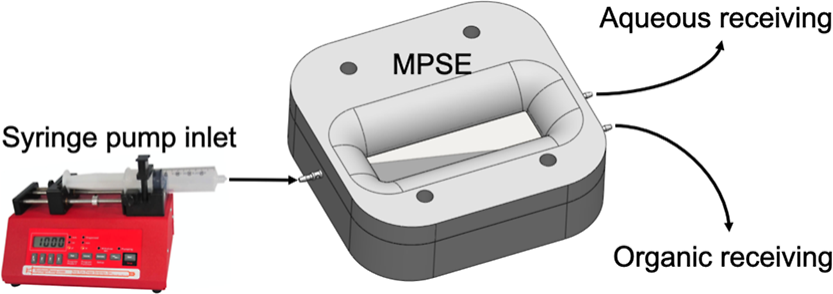
Slope-plate separation setup.

Two microscope glass slides (25 × 75 × 1 mm) were inserted into the device after washing with acetone and IPA and then dried to remove any potential airborne contaminations on the glass slides. We have conducted the separation experiment with both the uncoated and coated single-channel MPSE device using the water/hexadecane mixture by feeding an equal volume of pure DI water and hexadecane to the device using a Y-mixer and dual syringe pump (see Figure 4.) The purity was measured by dividing the designated liquid volume over the total volume coming out from the designated port. DI water was dyed blue to be differentiated from the hexadecane.

Separation efficiencies for two types of coatings as well as bare glass are compared using the single-channel device at a 10 mL/min flow rate as shown in Figure 5. With designated hydrophilic/oleophobic or hydrophobic/oleophilic coatings present on the glass slides, the separation efficiency increased significantly for both coatings compared to bare glass trials resulting in only 77 vol % DI water from the aqueous port and 63 vol % of hexadecane from the organic port. Glass slides coated with SAMs coating outperformed with 98 vol % DI water at the aqueous port and 88 vol % of hexadecane at the organic port.

**Figure 5:**
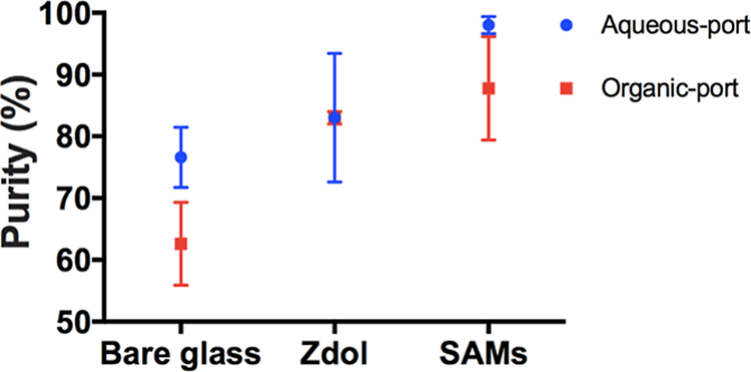
Separation efficiency comparison with and without different coatings using the single-channel device at a flow rate of 10 mL/min with dual BD syringes.

### Li Recovery with Ionic Liquid Solvent: Effect of Coatings

Ionic liquid has shown promise as a “greener” extraction solvent and been studied for lithium recovery extensively.^36^ A previous study^37^ has shown that an ionic liquid, i.e., 1-butyl-3-methylimidazolium bis­(trifluoromethylsulfonyl)­imide ([BMIM]­[NTF_2_]), with tri-*n*-butyl phosphate (TBP) as the extractant, can achieve a single extraction efficiency of Li as high as ∼92% under optimal conditions. We have investigated a very similar IL, 1-ethyl-3-methylimidazolium bis­(trifluoromethylsulfonyl)­imide ([EMIM]­[NTF_2_], as the extraction solvent for Li extraction using our MPSE technology in this study.

We conducted bulk lithium extraction from model saline solution (2.02 g/L Li^+^ ion in DI water) using a mixture of [EMIM]­[NTF_2_] and TBP (10%[EMIM]­[NTF_2_]/90%TBP) and achieved a single extraction efficiency of ∼84% for Li, as indicated by inductively coupled plasma mass spectrometry (ICP–MS) analysis. We then conducted lithium extraction from model saline solution (2.02 g/L Li^+^ ion in DI water) using a mixture of [EMIM]­[NTF_2_] and TBP (10%[EMIM]­[NTF_2_]/90%TBP) with our single-channel slope-plate MPSE device without any coating using an organic-to-aqueous (O/A) ratio is 1:1. In the MPSE setup, both syringes were loaded with 50 mL of the organic mixture (ionic liquid + TBP) and 50 mL of model brine. An inlet flow rate of 5 mL/min was applied to enhance the separation efficiency. Interestingly, as shown in Figure 6, 72 vol % of the organic substances, with a total volume of 36 mL, was discharged from the aqueous port, and 63 vol % of the water phase, with a total volume of 57 mL, was discharged from the organic port.

**Figure 6:**
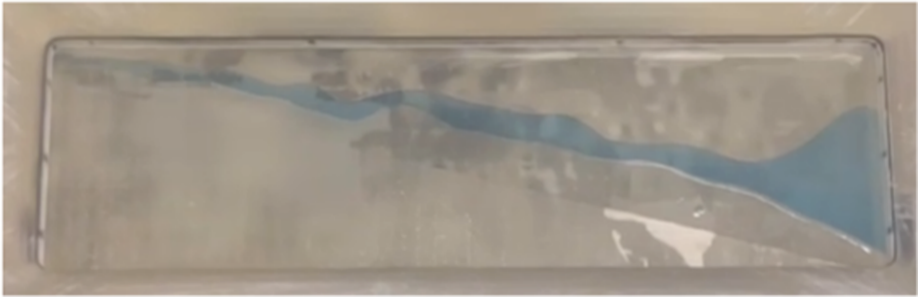
Separation interface using MPSE conducting Li extraction using EMIM-NTF_2_, TBP, and saltwater brine.

To understand the underlying mechanism of the poor separation, we conducted a pendant drop experiment to measure the interfacial tension of various liquid mixture components as shown in Table 1. The interfacial tension of TBP-IL and salt water is only ∼8.8 mN/m. Since the driving force of the separation is proportional of the interfacial tension, this might explain the unexpected the separation behavior. Since the interfacial tension between the IL and the salt water is only ∼13.6 mN/m, the potential to enhance the separation by adding more IL might be limited.

**Table 1:** Interfacial Tension Measurements

droplet	phase	interfacial tension (mN/m)
TBP/IL	salt water	8.8 ± 2.6
saline water	pure TBP	3.0 ± 0.1
IL (EMIM-NTF_2_)	saline water	13.6 ± 0.3

In order to optimize the separation, we applied coatings with the desired wettability on the glass slides. It is expected that the coatings will enhance the contact angle gradient and, thus, the capillary force gradient. Since the capillary force gradient is the driving force for the phase separation, coatings are expected to improve the separation efficiency. Two types of coatings were tested. The first one is a hydrophilic/oleophobic perfluoropolyether (PFPE) Zdol coating applied on the aqueous port. The second one is oleophilic/hydrophobic self-assembly monolayers (SAMs) with hydrocarbon segments, which is applied on the organic port. Table 2 summarizes the separation results of different coatings. Compared to bare glass slides, both surface coatings significantly improve the separation efficiency. Between the two coatings, Zdol glass slides outperformed the SAMs, with an aqueous port purity of 85% and an organic port purity of 67%.

**Table 2:** Effect of Coatings on MPSE Separation

coatings	flow rate	water-port purity	water-port *V*_total_ (mL)	oil-port purity	oil-port *V*_total_ (mL)
none (Bare glass)	5 mL/min	28%	36	37%	57
Zdol-only	5 mL/min	85%	27	67%	67
SAMs-only	5 mL/min	61%	33	67%	57

While the coatings have been shown to enhance MPSE separation of aqueous and organic phases, further efforts are required to optimize device-level lithium recovery using ionic liquids as extraction solvents. In addition, the durability of the coatings under prolonged exposure to the liquid phases remains to be systematically evaluated in future work.

## Conclusion

In the current work, a single-channel slope-plate modular (and membraneless) phase separation and extraction (MPSE) device was fabricated using 3D printing. To facilitate surface modification and improve interfacial control, the slope plate was designed with an insertable glass slide, allowing for versatile coating applications. Self-assembled monolayers (SAMs) and perfluoropolyether (Zdol) coatings were applied to the glass substrate to tailor the surface wettability and enhance the phase separation performance. The separation behavior of a model biphasic liquid system, water, and hexadecane was systematically studied using the single-channel MPSE device. Results show that both SAM and Zdol coatings significantly increase the separation efficiency compared with the uncoated plate. Building upon the model system, lithium extraction from a simulated saline solution was investigated using 1-ethyl-3-methylimidazolium bis­(trifluoromethylsulfonyl)­imide ([EMIM]­[NTf_2_]) as the ionic liquid (IL) extractant. The experiments demonstrated that the ionic liquid effectively extracted Li^+^ from the aqueous phase in bulk extraction. However, due to the intrinsically low interfacial tension between the IL and the aqueous phase, separation of the two liquids with a slope plate posed a significant challenge. The application of surface coatings proved to be an effective strategy to mitigate these issues. Overall, the integration of tailored surface chemistry with the slope-plate MPSE design offers a promising route to enhance the efficiency and controllability of ionic liquid-based extraction systems for lithium recovery from saline resources.

## Experimental Section

### Materials

Acetone (minimum 99.5%) and 2-propanol (isopropyl alcohol minimum 99.5%) certified ACS were purchased from Fisher Chemical. Deionized water produced by a Milli-Q system (with a resistivity of 18.2 MΩ·cm) was used in all the cleaning and immersion experiments. Concentrated sulfuric acid (95–95%) certifies ACS was purchased from Fisher Chemical, and hydrogen peroxide (30%) and sodium dichromate dihydrate (99.5%) were purchased from Sigma-Aldrich. For SAMs coating, octadecyltrimethoxysilane was purchased from Sigma-Aldrich and used as supplied. Hydrochloric acid was purchased from Fisher Scientific and used as supplied. Ethanol (200 proof) was purchased from the Dietrich School Scientific Stockroom of the University of Pittsburgh. The PFPE polymer, Zdol, was obtained from Solvay Solexis Inc. 2,3-Dihydrodecafluoropentane, commercially known as Vertrel XF, was purchased from Miller Stephenson Chemical Co., and it was used as the solvent for preparing Zdol solutions. All chemicals were used as supplied. Glass slides in the coating procedure were plain microscope slides (25 × 75 × 1.0 mm) purchased from Fisher Scientific and were used as supplied. To prepare the organic phase for separation, [EMIM]­[NTF_2_] (98%, Alfa Chemistry) and TBP (98%, Thermo Scientific) were mixed at a volumetric ratio of 10:90 (10 vol % [EMIM]­[NTF_2_] and 90 vol % TBP) until homogeneous. The model saline solution (2.02 g/L Li^+^ in DI water) was prepared by dissolving 12.34 g of LiCl (99%, Thermo Scientific) in DI water and diluting to a final volume of 1 L.

### Coating Procedure

#### SAMs

To remove any impurities from the glass slides, the following cleaning procedures were used every time: 10 min sonication in acetone followed by 10 min sonication in IPA solutions. To dry off the glass sample and remove any leftover impurities, glass slides were put in a vacuum oven for at least 30 min. Once these steps were completed, our glass slides are ready to be used in the next steps. Acid treatment solution was prepared by a mixture of sodium dichromate, sulfuric acid, and hydrogen peroxide and was mixed well until all sodium dichromate crystals dissolve. The solution turns from brown to dark green in the process and foams constantly due to formation of gas.^38^ The temperature of the acid solution was kept at 70 °C in a silicon oil bath and well mixed on the hot plate under the hood. Cleaned glass slides were immersed in an acid solution for around 1.5–2 h. The main purpose of this step is to create the hydroxyl groups on the glass surface to enable better attachment of Si–O–Si during the SAMs application. DI water washing steps were followed in order to wash off any remaining acid off the glass surface. This step was repeated 5 times to achieve successful acid solution removal. Glass slides were out in the oven at 100 °C for at least 30 min to dry the glass surfaces. Once dry, glass slides were immersed in SAMs solution of chloro­(dodecyl)­dimethylsilane, ethanol, water, and 0.1 M hydrochloric acid (1, 88, 9.5, 1.5 w % respectively) that was well mixed for at least 2 h and at 65–70 °C. Glass slides were immersed in SAMs solution for 10 min 4 times, drying in between at room temperature—under the hood. Oven heating overnight was the last step in the SAMs on the glass slide application process (to remove any volatile/loosely attached compounds). The schematic representation of surface preparation and coating is shown above.

#### Zdol

Glass slides were precleaned as mentioned above, undergoing acetone and IPA sonication wash, followed by vacuum oven drying. Additionally, they were treated by UV/Ozone under room temperature (∼24 °C) in ambient air for 10 min within a BioForce Nanosciences UV/Ozone Procleaner which emits a high-intensity UV light (110VCA, 50/60 HZ, 0.5A and 1 PH) with 185 and 254 nm wavelengths. One g/L Zdol solution was prepared by mixing 1 g neat Zdol and 1 L Vertrel XF, and glass slides were immersed and removed from the solution at a constant speed of 1 mm/s, with an immersion time of 20 s.

### Characterization of Coatings

The contact angles were measured by using a VCA Optima contact angle system. During the measurement, the liquids were dispensed through the needles onto the substrate, and the droplet images on the substrate were captured by a charge-coupled device (CCD) camera. The vendor-supplied software can calculate the contact angle values through the drop shapes. CA values were measured using water/hexadecane droplets of 1 μL in volume gently placed on the surface, after which their shape was evaluated by using the software. CA data presented were the average of at least five measurements on various surface locations of each sample.

The thickness of the SAMs and the Zdol coating on glass surfaces were measured by ellipsometry using a J.A. Woollam alpha SE spectroscopic ellipsometer at the incident angles of 65°, 70°, and 75°. Before glass slides were coated, the native oxide thickness was first determined for bare glass surfaces. The optical constants of the bare glass surfaces were determined using the B-Spline model. The thickness of the coating on the glass surfaces was determined using the Cauchy dispersion model.^39^ The film thickness measured by ellipsometry is an average of at least 5 measurements within the coated areas.

Interfacial tension was measured using pendant drop analysis with a VCA Optima XE system and its associated software. A ∼2 μL droplet of the test liquid was suspended from the tip of a needle submerged in the corresponding liquid phase. The droplet shape, influenced by the interfacial tension and gravity, was used to calculate the interfacial tension through numerical fitting of the drop contour via the Young–Laplace equation.

### Fabrication of the Slope Plate

The single-channel slope plate device is fabricated by a desktop stereolithography (SLA) 3D printer (Form 2, Formlabs) using clear photopolymer resin (Clear Resin 1L, Formlabs). 3D printing was performed on a platform with a maximum 145 × 145 × 145 mm^3^ building volume. CAD-designed modules exported as solid stereolithography files (STL) were imported to the slicing software (Preform, Formlabs) to be uploaded to the 3D printer. Preprogrammed settings in open mode at room temperature with a 100 μm layer thickness were selected to initiate printing tasks. Postprocessing includes washing the parts inside a washer (Form Wash, Formlabs) containing pure isopropanol (IPA) for 20 min followed by UV irradiation in a postcuring machine (Form Cure, Formlabs) for 60 min at 60̊C to enhance mechanical properties.

### Inductively Coupled Plasma Mass Spectrometry (ICP–MS) Analysis

Lithium ion concentration in the sample solution was determined using ICP–MS, with the Li^+^ ion signal monitored at a wavelength of 670.78 nm. The sample was first diluted 200 times with an appropriate solvent to achieve the desired concentration range for analysis. A total of 5 mL of the diluted sample was introduced into ICP, where the sample was atomized and ionized. The resulting lithium ions were then directed into the mass spectrometer and detected based on their mass-to-charge ratio (*m*/*z*). The Li^+^ concentration (mg/L) was calculated by comparing the signal intensity at 670.78 nm to that of a calibration curve constructed by using known lithium standards.

## Supplementary Material



## Notes

Coating geometry for both Zdol and SAMs coatings and photo of the slope-plate separation setup (PDF)
