# Supplementary material for: Toward Lithium Recovery Using Modular (and Membraneless) Phase Separation and Extraction (MPSE) Technology with Ionic Liquid (IL) Solvents: Effect of Coatings
Source: Langmuir. 2026 Mar 28;42(14):9861–7. doi: 10.1021/acs.langmuir.5c06615 (PMC13085800; doi:10.1021/acs.langmuir.5c06615)
Supplement: Supplementary file 1 [file la5c06615_si_001.pdf]

Supporting Information: **Towards Lithium Recovery Using Modular (and Membrane-less) Phase Separation and Extraction (MPSE) Technology With Ionic Liquid (IL) Solvents: Effect of Coatings**

Aigerim Baimoldina[1#], Fan Yang[1#], Yihan Song[1#], Jad George Touma[2], Francis Chukwunta[2], Matthew Coblyn[2], Cliff Kowall[3], Goran Jovanovic[2] and Lei Li[1*]

1: Department of Chemical & Petroleum Engineering, University of Pittsburgh, Pittsburgh, PA 15261, USA

2: School of Chemical, Biological and Environmental Engineering, Oregon State University, Corvallis, OR 97331, USA

3: Consulting Engineer, 4502 Liberty Road, South Euclid, Ohio 44092, USA

*: Corresponding author (Email: lel55@pitt.edu)

[#]: These authors contribute equally to the work

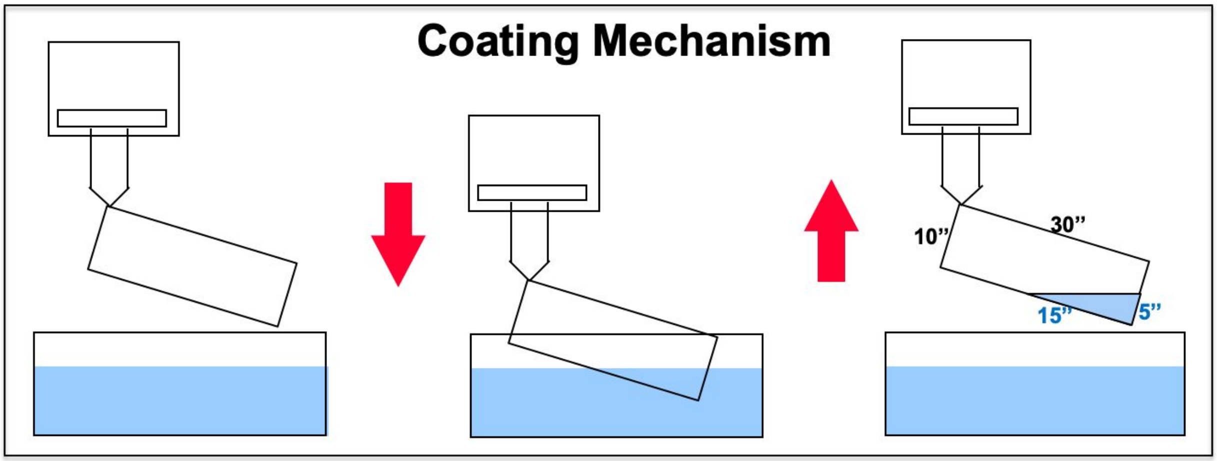

Figure S1: Coating geometry for both Zdol and SAMs.

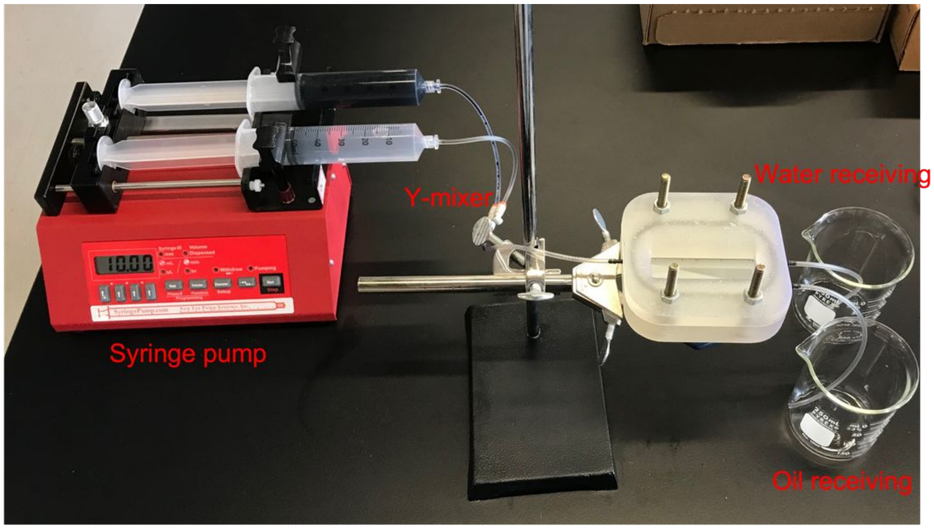

Figure S2. Photo of Slope-Plate Separation setup
